# Structure and phylogeography of two tropical predators, spinner (*Stenella longirostris*) and pantropical spotted (*S. attenuata*) dolphins, from SNP data

**DOI:** 10.1098/rsos.171615

**Published:** 2018-04-25

**Authors:** Matthew S. Leslie, Phillip A. Morin

**Affiliations:** 1 Scripps Institution of Oceanography, University of California San Diego, mail-code 0208, 9500 Gilman Dr., La Jolla, CA 92093, USA; 2 Southwest Fisheries Science Center, National Marine Fisheries Service, NOAA, 8901 La Jolla Shores Dr., La Jolla, CA 92037, USA

**Keywords:** pelagic dolphins, single nucleotide polymorphisms, conservation genomics, genotyping-by-sequencing

## Abstract

Little is known about global patterns of genetic connectivity in pelagic dolphins, including how circumtropical pelagic dolphins spread globally following the rapid and recent radiation of the subfamily delphininae. In this study, we tested phylogeographic hypotheses for two circumtropical species, the spinner dolphin (*Stenella longirostris*) and the pantropical spotted dolphin (*Stenella attenuata*), using more than 3000 nuclear DNA single nucleotide polymorphisms (SNPs) in each species. Analyses for population structure indicated significant genetic differentiation between almost all subspecies and populations in both species. Bayesian phylogeographic analyses of spinner dolphins showed deep divergence between Indo-Pacific, Atlantic and eastern tropical Pacific Ocean (ETP) lineages. Despite high morphological variation, our results show very close relationships between endemic ETP spinner subspecies in relation to global diversity. The dwarf spinner dolphin is a monophyletic subspecies nested within a major clade of pantropical spinner dolphins from the Indian and western Pacific Ocean populations. Population-level division among the dwarf spinner dolphins was detected—with the northern Australia population being very different from that in Indonesia. In contrast to spinner dolphins, the major boundary for spotted dolphins is between offshore and coastal habitats in the ETP, supporting the current subspecies-level taxonomy. Comparing these species underscores the different scale at which population structure can arise, even in species that are similar in habitat (i.e. pelagic) and distribution.

## 1. Introduction

High dispersal potential in marine organisms is thought to promote connectivity and evolutionary stasis in geographically distant populations [1]. Circumtropical marine species, however, face limitations to dispersal in the form of continents, cold water masses at higher latitudes, and open-ocean expanses, among others [2]. In circumtropical reef fishes, several major barriers to gene flow help shape various patterns of population genetic structure. These barriers include the open east Pacific basin, the Isthmus of Panama, the broad mid-Atlantic, and the Indo-Pacific barrier formed by the Indo-Malay Archipelago [3,4]. Ephemeral barriers may be present during some periods of deep time, but not others. This is almost certainly the case with the Benguela barrier of southern Africa, reflected in an Atlantic and Indo-Pacific connection in some species (summarized by Bowen *et al.* [3]). Pelagic predatory fishes with circumtropical distributions may lack populations structure between disparate ocean basins [5,6], show low levels of structure [7], show patterns of sex-biased gene flow [8], or reflect patterns of structure akin to reef-associated fishes that appear to be driven (at least in part) by the above barriers to gene flow [9]. Generalizations across pelagic population structure of predatory fishes are difficult to draw because of the complex mosaic of factors that contribute to form the patterns observed, including demography, vagility, migration, extinction, recolonization, etc.

Cetaceans are capable of long-distance movements, presumably increasing the tendency towards connectivity and stasis via gene flow. Davies [10], postulated that the Indo-western Pacific was a ‘warm water core’ for circumtropical delphinids. His reasoning was thus: because the Indo-west Pacific was buffered from cold-water intrusion during global cool periods, it presented a refuge for tropical dolphins. He further asserted that the Panamanian Isthmus, the continent of Africa and the east Pacific basin were significant barriers to these tropical species. These putative population boundaries [10] would drive population genetic structure and decrease global homogeneity. Despite Davies' inability to test his ideas, some of the barriers he postulated are similar to those Rocha *et al*. [4] describe for reef-associated fishes. Davies [10] did not include an Indo-Pacific boundary for tropical cetaceans.

Perrin [11] describes the southern tip of Africa as a ‘species gate’ occasionally opening during warmer geological periods to allow immigration of some cetacean populations from the Indian Ocean to the Atlantic. He highlights the unlikely possibility of the ‘gate’ being a two-way immigration corridor because of the very strong east-to-west currents that wrap southern Africa. Noting both the similarities and the interesting differences in dolphin taxa and communities between the Indian and Atlantic, Perrin believed that connection between these two ocean basins was relatively common over recent evolutionary time scales, but again only in one direction.

Unfortunately, testing hypotheses about populations in pelagic cetaceans is difficult given the logistical hurdles of sample collection from remote open ocean habitats [12]. Unlike coastally distributed cetaceans, carcasses of dead pelagic species rarely wash ashore post-mortem, making sample acquisition very unlikely without lengthy (and expensive) research expeditions or (as was the case in the ETP) a large network of fisheries observers collecting samples from animals caught and killed as bycatch. Because of these limitations, our knowledge about global genetic connectivity in pelagic dolphins is limited [12].

**Figure 1.**
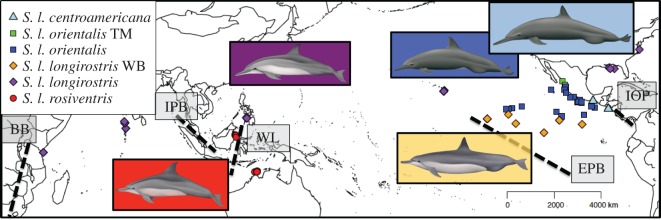
Sampling localities for spinner dolphins. Pale blue triangles indicate Central American spinners. Green squares are the proposed Tres Marias spinners. Orange diamonds indicate whitebelly spinners, a proposed intergrade between the pantropical or Grey's spinner (*S. l. longirostris*) indicated by purple diamonds, and the eastern subspecies (*S. l. orientalis*) shown as blue squares. Red circles indicate dwarf spinner dolphin (*S. l. roseiventris*) sampling locations. Potential genetic boundaries are shown in dashed black lines include: Isthmus of Panama (IOP), eastern Pacific basin (EPB), Indo-Pacific barrier (IPB), Benguela barrier (BB), Wallace's Line (WL). Illustrations by Uko Gorter (www.ukogorter.com/).

**Figure 2.**
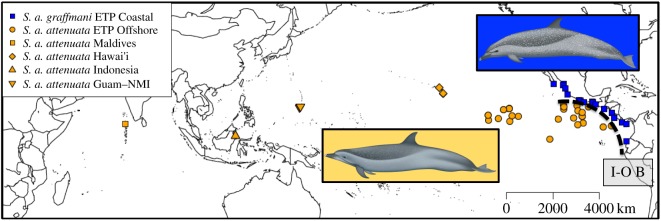
Sampling localities for pantropical spotted dolphins. Sampling localities for coastal spotted dolphins (*S. a. graffmani*) are depicted with blue squares, and pantropical spotted dolphins (*S. a. attenuata*) are shown in yellow. The Hawaiian population of pantropical spotted dolphins is shown in yellow diamonds whereas inverted yellow triangles show Guam and Northern Mariana Islands (NMI). Finally, Indonesian samples are labelled with standard yellow triangle and yellow squares indicate samples form the Maldives. Potential inshore–offshore genetic boundary (I-O B) is shown in black dashed line. Illustrations by Uko Gorter (www.ukogorter.com/).

Two circumtropical cetaceans, the spinner dolphin and spotted dolphins (*S. longirostris* and *S. attenuata*, respectively) present an opportunity to test these phylogeographic hypotheses. Both species exhibit intraspecific morphological variation (figures 1 and 2, respectively) that justifies the designation of multiple subspecies [13,14]. Spinner dolphins are divided globally into four subspecies: (i) the nominate ‘pantropical’ subspecies (*S. l. longirostris*) is distributed throughout the tropics with the exception of the eastern tropical Pacific (ETP), (ii) the Central American spinner dolphin (*S. l. centroamericana*), a coastal endemic subspecies in the ETP [13], (iii) the eastern spinner dolphin (*S. l. orientalis*), an offshore (pelagic) endemic subspecies in the ETP [13], and (iv) a dwarf spinner dolphin subspecies in Southeast Asia (*S. l. roseiventris*) [14]. Finally, a zone of introgression exists between the pantropical spinner dolphin subspecies of central and western Pacific and the eastern offshore subspecies of the eastern Pacific. The spinner dolphins in this area are known as ‘whitebelly’ spinner dolphins and exhibit traits intermediate to pantropical and eastern spinner dolphin subspecies [13].

These divisions were initially described using morphometric analyses [13,15], but have been confirmed genetically as well [16–18]. Andrews *et al*. [16] conducted a multi-locus phylogeographic study of global spinner dolphin populations. Using two mtDNA loci, one autosomal intron and three Y chromosome markers, they found low divergence among the subspecies and sampling areas. In addition, they found fixed differences between Y chromosome haplotypes from the eastern and Central American spinner dolphins subspecies compared to the pantropical and dwarf subspecies. Interestingly, this region of the Y chromosome was found to be polymorphic in the whitebelly [16]. This supports the hypothesis of hybrid origin of the whitebelly form. The result of this study showed weak concordance between genetic divergence and phenotype that suggested to the authors ‘porous’ genetic boundaries between phenotypically divergent groups. They further suggest that neutral genes are moving freely between groups, but genes subject to divergent natural selection have restricted transmission. The nuclear DNA comparison was with only one locus, however. Leslie & Morin [17], using a large single nucleotide polymorphism (SNP) dataset, found separation between all ETP groups using nuclear SNPs, but provided no global context for these differences. Leslie *et al.* [18] also found separation among these ETP groups using whole mitochondrial sequence data, but to a lesser degree. Aside from Andrews *et al*. [16], all these studies lacked global context as they focused exclusively on the ETP and Hawai‘i.

Pantropical spotted dolphins are divided into two subspecies globally: (i) the nominate subspecies (*S. a. attenuata*), and (ii) a coastal endemic subspecies in the ETP (*S. a. graffmani*) [19,20]. These designations were based on extensive analysis of cranial measurements, but have largely been confirmed by genetic data, including microsatellites [21], nuclear SNPs [17] and mitochondrial genomes [18]. These genetic studies lacked global context as they focused exclusively on the ETP and Hawai‘i. For pantropical spotted dolphins there has never been a global analysis of population structure or phylogeography using genetic data.

Using broader sampling and novel sets of more than 3000 nuclear SNPs, we expand upon recent studies [17,18] to test a series of hypotheses of oceanic boundaries for both pantropical species. First, for spinner dolphins, we examine the hypothesis of a single Atlantic + Indo-Pacific subspecies lineage that covers the broadest geographical range (from the central Atlantic Ocean to the western Pacific Ocean), separate from two endemic ETP subspecies. Second, we test whether the endemic ETP spinner dolphin subspecies are most divergent from the western Atlantic population due the uplift of the Isthmus of Panama (approx. 3.2 Ma). The spinner dolphin lineage originated about 3.14 Ma (2.02–4.47 Ma) according to molecular divergence dating [22]. The close timing of this origin with the uplift of Panama would suggest that there has not been a Pacific–Atlantic connection for most of the history of this species. Third, based on distributional proximity and morphological similarities between the two ETP endemic spinner dolphin subspecies [13,23], we hypothesize that the eastern spinner dolphin is more closely related to the Central American subspecies than to the pantropical subspecies. This pattern would result from the strong boundary formed by unsuitable habitat (low productivity, deep thermocline) in the east Pacific basin. In addition, we hypothesize that the whitebelly spinner will be difficult to place phylogeographically due to its putative mixed ancestry [16]. Finally, we test the hypothesis that the dwarf spinner dolphin is a distinct lineage from the Atlantic + Indo-Pacific subspecies lineage. There is no evidence from morphology or other data of spinner dolphins having a substantial boundary at the Indo-Malay Archipelago or at Wallace's Line, a biogeographic barrier that separates Asia from Australia [24].

For the spotted dolphins, our null hypothesis is two lineages: one including all populations of nominate pantropical subspecies (including the *S. a. attenuata* inhabiting offshore waters of the ETP), and another lineage that is exclusively the ETP coastal subspecies (*S. a. graffmani*) [19–21]. This hypothesis is based on cranial and external similarities in pantropical spotted dolphins around the globe, to the exclusion of the coastal spotted dolphin in the ETP. It is supported by a microsatellite study [21] that showed differentiation between pantropical offshore spotted dolphins and the coastal subspecies (with the exception of some coastal populations of Mexico that showed connection with the offshore subspecies). This scenario is counter to Davies [10] in that it does not invoke the east Pacific basin as a barrier, but rather an inshore–offshore barrier in the ETP as is present in some other delphinids in other parts of the globe (i.e. genera *Tursiops* and *Delphinus*) [25]. The spotted dolphin lineage purportedly diverged from other dolphins at 2.75 Ma (1.60–3.97), after the uplift of Panama [22]. Unfortunately, we were not able to include Atlantic specimens in our analysis to test the Panamanian Isthmus as a biogeographic boundary.

Analysing data from more than 3000 bi-allelic markers enabled us to test population structure hypotheses using relatively small samples sizes, such as those available for these animals from remote areas around the globe. Willing *et al*. [26] simulated population structure tests under different scenarios of sample size and number of SNP loci. They showed that sample size can be reduced to as small as four to six individuals when using a large number (greater than 1000) of bi-allelic markers without a loss in power to detect genetic differentiation using *F*-statistics. In fact, one of their simulations had characteristics very similar to our study (four individuals per population genotyped at 3000 loci). In this simulation the authors accurately detected genetic differentiation as low as *F*_ST_ = 0.01. Given the difficulty of obtaining larger sample sizes from many of the remote locations in this study, we have made use of large numbers of markers to obtain higher statistical power and precision for detecting population differentiation. Willing *et al*. [26] do make several assumptions in their simulations that are possibly violated in our study. These include even allele frequencies, equal sex ratios, an island model and no recombination. In addition, their simulations assume random sampling, which is difficult to achieve in nature. Linkage and selection could also impact results drawn from small samples. However, in terms of statistical power, the datasets we generated would be somewhat equivalent to 969–1733 microsatellite markers for spotted dolphins and 1023–1238 microsatellite markers for spinner dolphins (based on empirical and simulated datasets from Morin *et al.* [27], Narum *et al.* [28] and Smith *et al.* [29]).

## 2. Material and methods

### 2.1. Sample collection and DNA extraction

Skin samples were collected from spotted dolphins and spinner dolphins via biopsy dart [30] on research cruises, from specimens taken as bycatch in the tuna purse-seine fishery, or from stranded or beachcast individuals. Biopsies were collected in accordance to the best practices of the Society for Marine Mammalogy (https://www.marinemammalscience.org/about-us/ethics/marine-mammal-treatment-guidelines/), according to the permissible sampling techniques as stated on the project-specific Marine Mammal Protection Act (MMPA) Permit, and according to internal IACUC-approved methods.

Spinner dolphin samples collected on research cruises in the ETP were assigned to a population based on the external morphology of the majority of animals in the school. This approach was taken because: (i) these often-large groups (greater than 1000 individuals) contained individuals exhibiting a range of morphology; only after observing the group for some time could observers classify it to population, (ii) researchers collecting biopsies from dolphins near the bow of the research vessel found it very difficult to confidently classify fast-swimming individuals at sea, and (iii) there is significant overlap in range; therefore, geography was not a reliable predictor of population identity. Samples were selected from areas where the eastern and whitebelly types are known to overlap, as well as from outside the overlap region (figure 1). The most experienced observers on the research cruise made the assessment of the type of the majority of the school prior to sampling, but there was probably some error involved. Unfortunately, there is no way to measure the accuracy of each sampling event. Spotted dolphins samples (figure 2) were assigned to subspecies and populations based on the geographical location of the sampling site. In areas where the two ETP subspecies overlap, spotted dolphin samples collected from research cruises were assigned to populations based on external morphology [31,32].

Tissue samples were preserved in salt-saturated 20% DMSO or 70% ethanol and stored frozen at −20°C, or frozen at −80°C with no preservative. DNA was extracted using silica-based filter membranes (Qiagen, Valencia, CA) or by NaCl precipitation [33]. DNA was quantified using Pico-Green fluorescence assays (Quant-it Kit, Invitrogen, Carlsbad, CA) using a Tecan Genios microplate reader (Tecan Group Ltd, Switzerland). DNA quality was assessed by electrophoresis in 1% agarose gel; only high-molecular weight extracts were used.

Sequencing libraries were constructed using a ‘genotyping-by-sequencing’ protocol [34] as previously described [17] using the PstI enzyme. Library preparation and multiplexed sequencing on an Illumina *HiSeq* 2000/2500 (100 bp, single-end reads) were completed at the Cornell University Institute of Biotechnology's Genomic Diversity Facility (http://www.biotech.cornell.edu/).

### 2.2. Filtering, assembly, SNP discovery and genotyping

A subset of the samples and resulting data have previously been used to investigate population structure in the ETP [17]. For the ETP samples and additional samples added for this global analysis, data filtering, assembly and genotyping were as described by Leslie & Morin [17]. Filtered data for this paper are available in an R environment, Dryad: http://dx.doi.org/10.5061/dryad.h9q13 [35].

### 2.3. Diversity estimates and populations structure analyses

Per-population heterozygosity was calculated using the *strataG* [36] package in R [37]. We then estimated differentiation (*F*_ST_) for each pairwise combination of populations [38–40]. Point estimates and permutation tests (1000 repetitions) were generated using the *strataG* package in R [36].

We also directly tested hypotheses of population differentiation using multivariate analyses, specifically the discriminant analysis of principal components (DAPC) in the R package *Adegenet* [41]*.* DAPC calculates principal components and then estimates a centroid and measures the variance for predefined populations. The discriminant analysis tests the probability of each individual falling in the space of each of the populations based on the ‘geometric space’ created by the centroid and variation. Before conducting the DAPC analyses, we examined the cumulative variance explained by the eigenvalues for the full range of principal components.

Because of the size and variability of these datasets, spurious ad hoc solutions might be found. These include, but are not limited to, over-fitting (i.e. using too many principal components and thus resulting in large and unstable differences between populations). To assess if over-fitting was occurring, we calculated alpha-scores for each population and each dataset overall, simulated in *Adegenet* (simulated 10 times).

To complete the DAPC, we then constructed synthetic discriminant functions that represent linear combinations of the allelic data with the largest between-group variance and the smallest within-group variance. In all analyses, we kept only the first three eigenvalues, as they represented the vast majority of the information. Finally, we plotted the first two discriminant functions as two-dimensional scatters in R [37].

### 2.4. Phylogeographic analyses

Phylogeographic analyses were performed using *SNAPP*, a Markov chain Monte Carlo (MCMC) sampler for bi-allelic data used to infer phylogenetic trees [42]. Because of the high number of SNP loci for each individual and because phylogenetic analyses of large datasets are computationally intensive, the sample sizes for these analyses were reduced. Two samples were chosen at random from each putative population for spinner dolphins, and between one and seven were taken for spotted dolphins because of the lesser number of populations. Sample details are listed in electronic supplementary material, tables S1 and S2. Given the differences between populations (based on *F*_ST_ and DAPC), we did not replicate these analyses with different samples selected from each population.

*SNAPP* was implemented in the software package *BEAST 2* [43]. Prior to the analyses, datasets were converted using custom R scripts from the *strataG* format (*gtype*) to nexus format, input into *Beauti* (v. 2.3.1; [43]) and exported as .xml files. Forward and reverse mutation rates were estimated and chains were sampled every 1000 iterations. Coalescence rate was sampled throughout the MCMC. All other settings followed the default given in *Beauti*.

*SNAPP* log files were read into *Tracer* (v. 1.6.1; [44]) to evaluate the convergence of the MCMC analyses. This included assessing the overall quality of the analyses inferred by the trends and variance of the estimates of Bayesian posteriors and estimated sample size (ESS), and estimating the number of chains to remove as burn-in.

We used *DensiTree* (v. 2.01; [45]) to visualize and qualitatively analyse phylogeographic relationships and uncertainty using multiple trees. *DensiTree* displays the frequency of topologies as the colour of the trees presented. The most popular topologies are blue, the second most popular topologies are red and other topologies are green. *TreeAnnotator* (v. 2.3.1; [46]) was used to produce a consensus tree for the *SNAPP* analysis for each dataset. Burn-in for *TreeAnnotator* and *DensiTree* were set at 10%. We limited the posterior probability calculation for each node in the maximum clade credibility tree to those with greater than 0.5 posterior probability. Common ancestor heights were used for all consensus tree node heights. Finally, the consensus tree topology, posterior probability for each node, and theta for each branch were visualized in *FigTree* (v. 1.4.2; [47]).

Although, there are methods for inferring the location of the root [48] based on calculating the posterior probability of the root location, these options are not available for SNP data presently. Moreover, using outgroups in *SNAPP* can create long branches which make parameter estimation difficult and violate the assumptions of the Yule prior used in *SNAPP* (R. Bouckaert 2014, Personal Communication).^1^ Midpoint rooting often results in the root being assigned to the longest branch, which is the case in both of our species. This may not, however, reflect reality in terms of ancestry. Thus, we have tried to avoid making inferences dependent on the location of the root. It is also possible that phylogeographic patterns in nuDNA trees could represent shared ancestry, admixture (i.e. genetic exchange between populations) or a combination of both.

## 3. Results

The average read depth for spinner and spotted dolphin SNP genotypes was 36.8 and 31.71, respectively. To remove genotypes with high coverage possibly due to clonality, repetitive elements or gene duplications, those with coverage depth greater than 65.22 and 57.88 (the mean read depth plus >1.5 times the standard deviation) were removed from the spinner and spotted dolphin datasets, respectively. The final datasets for each species included 118 spinner dolphins and 75 spotted dolphins genotyped at 3340 and 3524 SNPs, respectively.

Summaries of the sample sizes and mean heterozygosity for spinner and spotted dolphins are found in tables 1 and 2, respectively. Mean heterozygosity across SNPs for all individuals within a population (*H*) is indicative of the overall genetic variation within the populations. The datasets for the two species were comparable in terms of overall heterozygosity: spotted dolphins = 0.2452 (s.d.: 0.1272); spinner dolphins *=* 0.2494 (s.d.: 0.1317).

**Table 1.** Population genetic summary statistics for spinner dolphins (*S. longirostris*) based on 3340 SNPs. *H* (s.d.) is the mean heterozygosity (and standard deviation) per site across all individuals in each population. Numbers of samples show for *n* (total), F (females), M (males) and U (unknown sex).

subspecies	populations	*n*/F/M/U	*H* (s.d.)
*centroamericana*	Central American	9/5/3/1	0.2601 (0.2116)
*centroamericana*	Tres Marias	12/6/6/0	0.2589 (0.1881)
*orientalis*	eastern	37/18/18/1	0.2766 (0.1600)
*longirostris*	whitebelly	14/7/7/0	0.2644 (0.1795)
*longirostris*	Hawai‘i	6/2/4/0	0.2220 (0.2296)
*longirostris*	Philippines	9/4/2/3	0.2472 (0.2024)
*longirostris*	Maldives	6/4/2/0	0.2532 (0.2279)
*longirostris*	Tanzania	3/0/0/3	0.2431 (0.2750)
*longirostris*	North Atlantic	4/0/3/1	0.2320 (0.2585)
*roseiventris*	Indonesia	6/0/0/6	0.2355 (0.2229)
*roseiventris*	Australia	12/1/11/0	0.1590 (0.1978)
	overall	118/47/56/15	0.2494 (0.1317)

**Table 2.** Population genetic summary statistics for spotted dolphins (*S. attenuata*) based on 3524 SNPs. *H* is the mean heterozygosity per site across all individuals in each population. Numbers of samples show for *n* (total), F (females), M (males) and U (unknown sex). NMI, Northern Marianas Islands.

subspecies	populations	*n*/F/M/U	*H* (s.d.)
*graffmani*	ETP coastal	27/11/14/2	0.2378 (0.1730)
*attenuata*	ETP offshore	32/22/7/3	0.2632 (0.1488)
*attenuata*	Hawai‘i	4/2/2/0	0.2148 (0.2503)
*attenuata*	Guam/NMI	8/0/0/8	0.2326 (0.2109)
*attenuata*	Indonesia	3/0/0/3	0.2274 (0.2097)
*attenuata*	Maldives	1/0/0/1	**
	overall	75/35/23/17	0.2452 (0.1272)

** = *H* could not be calculated for Maldives, as there was only one sample from this population.

The disparity in sample sizes between ETP populations and those from the global sampling made comparisons of heterozygosity within species difficult. However, in both spinner and spotted dolphin datasets, Hawaiian dolphins fell in the lower range. In addition, dwarf spinner dolphins from Australia exhibited the lowest mean heterozygosity measured in this dataset. Low heterozygosity could indicate smaller populations, inbred individuals, or poor sample quality that results in allelic dropout [49,50]. Finally, the eastern spinner and the offshore ETP spotted dolphin populations had high heterozygosity. High heterozygosity could indicate outbred individuals, large historical population abundance, or be an artefact of the higher sample sizes for these populations. ETP spinner and spotted dolphins are known to have historically high abundances. Gerrodette *et al*. [51] estimated population abundances based on data from a 2006 survey: eastern spinner (1 062 879; CV = 0.26), whitebelly spinner (734 837; CV = 0.61), northeastern offshore spotted (857 884; CV = 0.23), western/southern offshore spotted (439 208; CV = 0.29), coastal spotted (278 155, CV = 0.59). However, this is only a fraction of what they were prior to high levels of incidental bycatch, potentially one-third and one-fifth of historical abundance for spinner and spotted dolphins, respectively [52]. Despite this dramatic reduction, it is likely that high heterozygosity is the result of high historical abundance.

### 3.1. Population structure: ***F***_ST_

Pairwise tests of population differentiation based on allele frequencies showed high levels of differentiation in both species (tables 3 and 4) despite high dispersal potential of individual animals. Almost all the pairwise *F*_ST_ estimates for spinner and spotted dolphins were significantly different from zero. For spinner dolphins, two pairwise comparisons were not significantly different: Central American versus Tanzania and Maldives versus Tanzania. The non-significant estimates for comparisons involving Tanzania could be a result of the small sample size for that group (*n* = 3). The lowest point estimate for *F*_ST_ was between the eastern spinner subspecies and the Tres Marias population (*F*_ST_ = 0.0035), but all point estimates between ETP populations were low (i.e. *F*_ST_ < 0.022). For spotted dolphins, all pairwise comparisons were significantly different from zero (*p* < 0.05). The largest differences observed involved comparisons of the ETP coastal subspecies with populations from the central and western Pacific Ocean. Lower pairwise values were found between the populations of the pantropical subspecies, despite long distances between them. Pairwise statistics for comparisons with the Maldives were not included because only one sample from this population was genotyped.

**Table 3.** Pairwise population genetic differentiation statistics for spinner dolphins from 3340 SNPs. *F*_ST_ below diagonal and *p*-value above.

subspecies	populations	*n*	CA	TM	East	WB	HI	Phil	Mal	Tan	NA	Indo	Aus
Central America (CA)		9	—	0.0209	0.0009	0.0009	0.0009	0.0009	0.0009	0.0549*	0.0020	0.0009	0.0009
Tres Marias (TM)		12	0.0084	—	0.0009	0.0009	0.0009	0.0009	0.0009	0.0060	0.0020	0.0009	0.0009
Eastern (East)		37	0.0119	0.0035	—	0.0009	0.0009	0.0009	0.0009	0.0010	0.0020	0.0009	0.0009
	whitebelly (WB)	14	0.0210	0.0113	0.0074	—	0.0009	0.0009	0.0009	0.0040	0.0019	0.0009	0.0009
	Hawai‘i (HI)	6	0.1036	0.0890	0.0793	0.0696	—	0.0009	0.0029	0.0171	0.0039	0.0009	0.0019
	Philippines (Phil)	9	0.0601	0.0467	0.0395	0.0219	0.0733	—	0.0009	0.0050	0.0039	0.0009	0.0009
	Maldives (Mal)	6	0.0507	0.0419	0.0322	0.0167	0.0709	0.0161	—	0.3166*	0.0059	0.0009	0.0009
	Tanzania (Tan)	3	0.0505*	0.0383	0.0293	0.0119	0.0812	0.0179	0.0003*	—	0.0285	0.0099	0.0020
	North Atlantic (NA)	4	0.0765	0.0661	0.0576	0.0518	0.1242	0.0707	0.0657	0.0619	—	0.0039	0.0029
	Indonesia (Indo)	6	0.0663	0.0580	0.0497	0.0279	0.0797	0.0177	0.0214	0.0195	0.0762	—	0.0009
	Australia (Aus)	12	0.2742	0.2567	0.2207	0.2236	0.2730	0.2172	0.2194	0.2566	0.3015	0.1698	—

* is *not* significantly different from zero.

**Table 4.** Pairwise population genetic differentiation statistics for spotted dolphins from 3524 SNPs. *F*_ST_ is below the diagonal and *p*-value is above. All comparisons are significantly different from zero (*p* < 0.05). Pairwise statistics for comparisons with the Maldives were not included because only one sample from this population had genotype data. NMI, Northern Mariana Islands.

subspecies	populations	*n*	ETP coastal	ETP offshore	Hawai‘i	Guam/NMI	Indonesia
ETP coastal		27	—	0.0009	0.0009	0.0009	0.0010
ETP offshore		32	0.0547	—	0.0009	0.0009	0.0020
	Hawai‘i	4	0.1717	0.0515	—	0.0039	0.0359
	Guam/NMI	8	0.1577	0.0387	0.0215	—	0.0039
	Indonesia	3	0.1727	0.0475	0.0391	0.0124	—

### 3.2. Population structure: DAPC

The distribution of genotypes for spinner and spotted dolphins was analysed using discriminant analysis of principal components (DAPC). Before conducting the DAPC analyses, we examined the cumulative variance explained by the eigenvalues for the full range of principal components. This test (run for a maximum of *k* = 20) indicated that there was no reason for keeping a small number of PCs (see electronic supplementary material, figures S1 and S2). To examine the breadth of possible reasonable results from the data, we conducted hypothesis testing using both the maximum recommended number of PCs and the ‘optimum’ number of PCs. The maximum number of PCs recommended by the developers of *Adegenet is n*/3, or 39 and 25 PCs for spinner and spotted dolphin analyses, respectively. We determined the optimal number of PCs using alpha scores. Alpha is the reassignment probability calculated using the given populations minus the reassignment probability for randomly permuted clusters, and indicates the optimum number of PCs (i.e. the highest mean Alpha across all simulations). For our datasets 7 PCs was the optimum for the spinner dolphins and 6 PCs for the spotted dolphin data (electronic supplementary material, figures S3 and S4).

**Figure 3.**
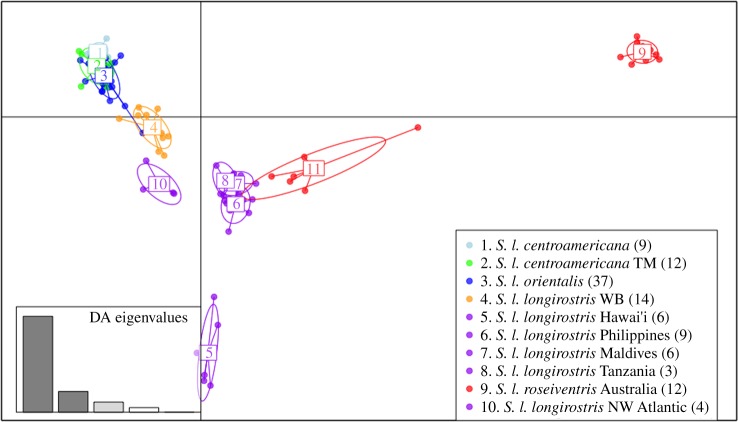
Genomic variation across individuals and populations of spinner dolphins. Scatter plot of individuals based on the first two eigenvalues (created from optimum nine principal components) of the DAPC. Ellipses represent 67% of the variation for each population. Inset shows the amount of variation represented by the DA eigenvalues. Points and ellipses are coloured by subspecies (red = *S. l. roseiventris*; purple = *S. l. longirostris*; dark blue = *S. l. orientalis*; light blue =*S. l. centroamericana*) and population (orange = whitebelly spinner; light green = Tres Marias Island spinner).

**Figure 4.**
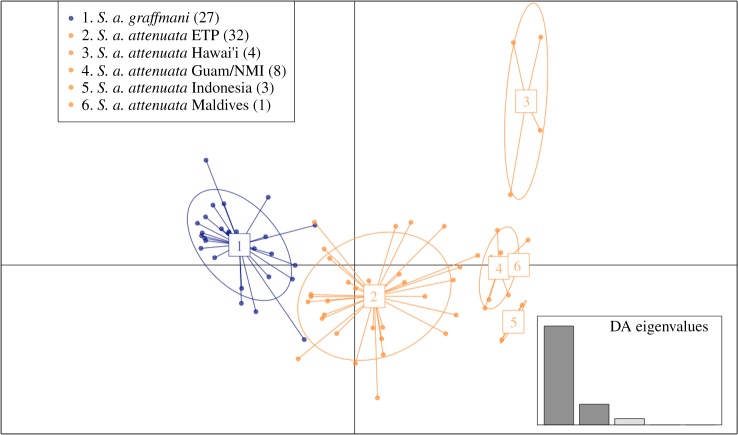
Genomic variation across individuals and populations of spotted dolphins. Scatter plot of individuals based on the first two eigenvalues created from the optimum number of principal components (9) of the DAPC. Ellipses represent 67% of the variation for each population. Maldives (6) is only one sample. Inset shows the amount of variation represented by the DA eigenvalues. Points and ellipses are coloured by subspecies (orange = *S. a. attenuata*; blue = *S. a. graffmani*).

Results differed little using the n/3 or alpha-estimated optimum number of PCs. The main difference was the ‘spread’ within and between populations. Using the optimum number of PCs (i.e. trying to avoid over-fitting), the populations are relatively tightly clustered with closely related populations overlapping (figures 3 and 4), whereas in the n/3 analysis there is more distance between the ellipses (electronic supplementary material, figures S5 and S6).

Genomic variation across spinner dolphin individuals and populations was represented well by the first two eigenvalues of the DAPC although one eigenvalue was clearly dominant (inset figure 3). This dominant eigenvalue (the horizontal) spread the genotypic data into two arms radiating from a core group. The tightly clustered ETP endemic subspecies (1–3) formed the distal end of one arm. Along that same arm, but opposite the ETP group and closer to the core groups, were the whitebelly (4) and North Atlantic spinners (10). The core group consists of overlapping ellipses of genotypes from geographically disparate groups: the Philippines (6), Maldives (7) and Tanzania (8). Radiating from the core opposite to the ETP + Atlantic arm are the two populations of the dwarf subspecies: Indonesia (11) and Australia (9). Indonesia is closer to the core, with its ellipse extending over some individuals from the core group. The Australia population (9) formed a tight cluster at the distal end of this arm—the farthest from all the other groups including the other population of dwarf spinners (11). Finally, the second eigenvalue separated Hawaiian (5) genotypes away from the core group in a separate and distant cluster.

As shown in the inset of figure 4, the first eigenvalue represents the majority of the variation in the spotted dolphin dataset. Coastal (1) and offshore ETP (2) subspecies samples show separate ellipses but with some individuals very close in genomic space. Hawaiian samples (3) occupy a distant PC space separate from all the other groups, but in a relatively large cluster. The Guam/NMI group (4) forms a tighter cluster near the offshore ETP group. The Indonesian samples (5) also form a tight distinct cluster near the Guam/NMI group. Finally, the single sample from the Maldives (6) overlaps with the Guam/NMI group.

### 3.3. Phylogeography

**Figure 5.**
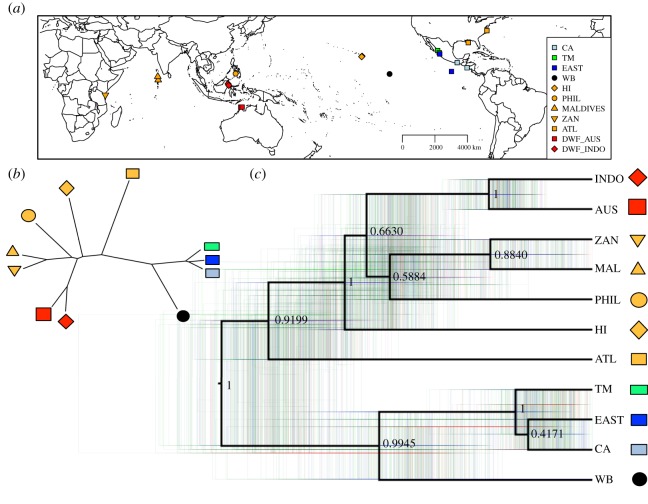
Bayesian species tree for spinner dolphins using 3340 SNPs. (*a*) A map representing the sampling localities for the reduced sample set used (*n* = 22) to decrease computation time for phylogeographic analysis (see electronic supplementary material, table S1). (*b*) The unrooted maximum clade credibility (MCC) tree. CA: Central America subspecies (*S. l. centroamericana*) is labelled with a light blue box. EAST: Eastern spinner dolphin subspecies (*S. l. orientalis*) is the dark blue box. WD: whitebelly spinner is the black circle. All other pantropical (*S. l. longirostris*) are labelled in orange: ATL = NW Atlantic; HI = Hawai‘i; ZAN = Tanzania (Zanzibar); PHIL = Philippines. Red populations are dwarf spinner dolphins from Indonesia (diamond = INDO) and northern Australia (square = AUS). (*c*) In black is the mid-point rooted MCC tree. The MCC tree is overlaid on all trees (coloured) from the analyses (blue trees are the most common, red are the next most common and green are the least common). Nodes are labelled with posterior probabilities.

**Figure 6.**
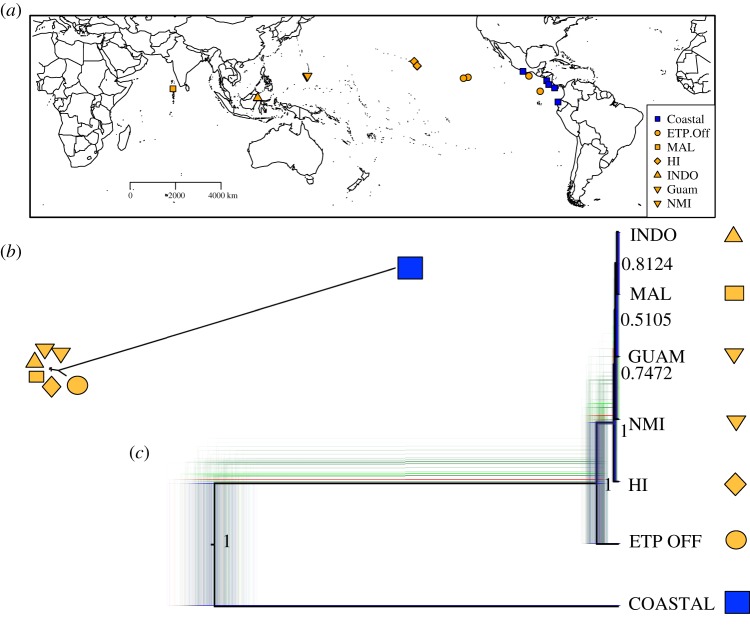
Bayesian species tree for spotted dolphins 3524 SNPs. (*a*) A map representing the sampling localities for the reduced sample set used (*n* = 22) to decrease computation time for phylogeographic analysis (see electronic supplementary material, table S2). (*b*) The unrooted tree MCC tree. The coastal subspecies (*S. a. graffmani*) is labelled with a blue box, while all other pantropical (*S. a. attenuata*) are labelled in orange. Offshore eastern tropical Pacific spotted dolphins are represented by an orange circle (ETP OFF); Hawai‘i is represented by an orange diamond (HI); Northern Marianas Islands (NMI) and Guam are represented by inverted triangles; Maldives (MAL) is labelled with a square and Indonesia (INDO) is labelled with a standard triangle. (*c*) In black is the mid-point rooted MCC tree. It is overlaid onto all trees, coloured as in figure 5.

The preceding has largely focused on population structure and not on relationships among different populations and processes leading to the patterns we observe today. To help visualize the results in an unbiased way, we present both unrooted and midpoint-rooted trees in figures 5 and 6.

Spinner dolphin phylogeographic topology was evaluated in *SNAPP* for a total of 401 000 chains. This was short of our desired >1M chains, but the sampler converged well during this amount of iterations (electronic supplementary material, figure S7). The developers of *SNAPP* suggest letting the chain run until the estimated sample size (ESS) is greater than 100, although they make clear that this is not an empirically tested threshold for stability. Our run resulted in an ESS for the posterior of 235, indicating adequate stability using this somewhat subjective criterion.

Spinner dolphins form two major, strongly supported (posterior probability = 1) clades of diversity (figure 5*c*). One clade consists of the eastern tropical Pacific Ocean groups, comprised of whitebelly, eastern, Central American and Tres Marias populations. Within this ETP clade, whitebelly spinners were well supported as the most basal lineage.

The second major clade contained samples from three ocean basins: North Atlantic, Indian Ocean, and central and western Pacific Ocean. The most basal lineage in this clade was the Atlantic Ocean spinner dolphins, followed by an Indo-Western Pacific clade with Hawai‘i as the most basal lineage. Relationships among the geographically disjunct Indo-Western Pacific groups were less well resolved, except for the strongly supported dwarf spinner dolphin clade.

The spotted dolphin dataset was analysed for a total of 1 M MCMC chains. The trace of the posteriors resulting from MCMC sampler showed minor variability (electronic supplementary material, figure S8) and the ESS was 67—indicating a possible need for additional MCMC iterations. Aside from some of the terminal nodes, the consensus tree was well supported (figure 6). The topology shows two very distinct major lineages: (i) the endemic coastal ETP subspecies, and (ii) a clade that included all other populations of the nominate pantropical subspecies (posterior = 1). Offshore ETP spotted dolphins were the most basal lineage of the pantropical clade, sister to a very shallow clade that included Hawai‘i, NMI, Guam, Maldives and Indonesia populations. Relationships between these Indian Ocean and western Pacific populations were poorly supported as indicated by the low nodal support values.

## 4. Discussion

Davies [10] proposed phylogeographic hypotheses for circumtropical cetaceans that included an Indo-western Pacific core and two satellite lineages stretching into the Atlantic to the west, and the eastern tropical Pacific to the east. Perrin [11] argued for a similar arrangement, but noted that the southern Africa barrier was probably only passable from east to west during warm regimes. Both ideas included barriers in east Pacific basin and southern Africa, even for these animals with high dispersal potential. Overall, our results support these biogeographic hypotheses in spinner and spotted dolphins, but with some interesting differences between the species. Because the unrooted phylogeographic analyses do not represent a sequence of events, only a pattern of relatedness, we have limited ability to infer concrete biogeographic events. Nevertheless, the pattern observed provides valuable insights for biogeographic hypotheses.

### 4.1. Spinner dolphins

For spinner dolphins, our analyses supported: (i) a geographically widespread Indo-western Pacific lineage, (ii) a divergent ETP lineage, comprised of closely related subspecies/populations, (iii) a whitebelly spinner dolphin related to the ETP group, (iv) a divergent Atlantic lineage sister to the Indo-western Pacific group, (v) an independent Hawaiian lineage, and (vi) a dwarf spinner dolphin lineage sister to the Indo-western Pacific group.

#### 4.1.1. Indo-western Pacific Ocean group

A geographically disparate set of three spinner dolphin populations in the Indo-western Pacific (that mostly differed in allele frequencies) overlapped in genetic DAPC space and formed a clade in the Bayesian phylogeographic analysis. We were surprised by the amount of similarity between these groups, that included western tropical Pacific (Philippines), western Indian Ocean (Tanzania) and Central Indian Ocean (Maldives). Separated by up to 5441 km, these populations have remained genetically similar. These results suggest that the Indo-Pacific boundary is not a barrier to genetic connectivity. Interestingly, Galver [53], using some of the Maldives samples we used in this study, found mtDNA haplotypes shared with the dwarf spinner populations, suggesting possible exchange between these groups. Our phylogeographic analysis of nuDNA resulted in a sister relationship between this Indo-western Pacific Ocean group and the dwarf subspecies. Although some of our populations have low sample sizes, simulations have shown the ability to precisely estimate *F*_ST_ to detect allele frequency differences when using a large number of genetic markers (i.e. greater than 1000), even from small sample sets (*n *> 4–6) [26]. The two pairwise comparisons that were not significant involved a population for which we had only three samples: Tanzania. This population showed non-significant differences when compared to two other groups (Maldives and Central America). It is probable that a connection exists between the more proximate populations—Tanzania and Maldives—but the *F*_ST_ results should remain preliminary given the sample size of only three [26]. Note that this connection was supported by the phylogeographic analysis and the DAPC results. The *F*_ST_ results that showed non-significant differences between Tanzania and Central America are probably anomalous as this connection was not supported by either the phylogeny or the DAPC results.

#### 4.1.2. Eastern tropical Pacific Ocean

The longest branch in our Bayesian species tree separated spinner dolphins in the eastern tropical Pacific Ocean from the rest of the global distribution of pantropical and dwarf subspecies (figure 5). This suggests that the eastern Pacific basin—an expanse of 4000–7000 km—is a formidable biogeographic barrier (and may have been since early in this species' history (see [54])). More specifically, however, the western portion of pelagic eastern Pacific (which contains a substantial population of spinner dolphins) is a very different habitat than that of the ETP [55]. This habitat differential may have resulted in selection pressures that drove (and retained) divergence between ETP spinners and those in the rest of the world.

Our modern conception of spinner dolphin relationships within the eastern Pacific Ocean (informed by morphology, distribution and genetics) says there are two separate evolutionary lineages (Central American (CA) and eastern) despite some ongoing genetic connection [13,16–18]. The CA and eastern subspecies are morphologically and genetically distinct from one another; however, the global view of spinner dolphin genomic diversity (figure 5) yields an interesting perspective on the ETP subspecies.

Our DAPC analysis showed CA, eastern and Tres Marias Island spinner dolphins in an overlapping cluster at one end of the DAPC space. Using a similar SNP dataset and the same DAPC analyses, Leslie & Morin [17] found separation between these ETP subspecies (and the Tres Marias population). *F*_ST_ estimates in that study and the present study support differences between these subspecies based on allele frequencies. However, viewed within the context of the overall global genetic diversity, the morphometrically and genetically distinct endemic ETP subspecies effectively occupy an overlapping ‘genetic space’. The two endemic subspecies of the ETP are joined by the shortest branches in our phylogeographic analysis as well; thus, despite being classified as independent subspecies, they are genetically the most similar of any pair of populations or subspecies in our dataset. The low support of nodes within the subspecies clade (posterior = 0.42) is not surprising, given how closely related they are thought to be [17]. These results support the hypothesis that the eastern Pacific basin and the Isthmus of Panama are major barriers to gene flow.

#### 4.1.3. Whitebelly spinner dolphin

The whitebelly (WB) spinner dolphins were near the ETP endemics in the DAPC results—about half way between these subspecies and the pantropical Indo-western Pacific group. This is what we would expect given the hypothesis that it is a hybrid swarm between eastern and central Pacific pantropical spinner dolphins. Contemporary bi-directional gene flow would ensure the whitebelly genome remains similar to both the pantropical and eastern subspecies. According to our phylogeographic analyses, the WB spinner dolphin is a sister lineage to the ETP endemic subspecies. Although our unrooted analysis limits our ability to strongly infer ancestry, if this is a case of strict ancestry, the similarities in morphology between WB and the pantropical subspecies are synapomorphies, and the novel traits of the eastern and Central American subspecies are apomorphies. Alternatively, the WB spinners could represent reconnection between subspecies separated and independent for some time and only recently interbreeding. Andrews *et al*. [16], in their genetic study of global spinner dolphin relationships, found ‘porous’ genetic boundaries between the ETP and pantropical subspecies. They suggest that neutral genes have moved freely between groups, but genes subject to divergent natural selection should show differences. Evidence for this was from a shared Y-chromosome haplotype in the eastern and Central American subspecies that was not found in pantropical or the dwarf subspecies. Interestingly, this region of the Y-chromosome was found to be polymorphic in the whitebelly form [16]. We did not separate neutral from selected loci, and therefore cannot speak to the contrasting patterns Andrews *et al*. [16] saw in different marker types with regard to ETP spinner dolphins.

We saw little evidence in this analysis (or *F*_ST_ estimates) of connection between the ETP spinner groups (including WB) and the Hawaiian pantropical spinners. Unfortunately, our sampling in the central Pacific was geographically sparse and may not have included the island-associate populations most closely related to the WB spinners. If there were going to be connections with the WB they might come from south of Hawai‘i. Adding data from these regions could be informative with regard to the level of connection between WB and the central Pacific populations. We recommend further testing for admixture in closely related ETP populations to examine the origin of the whitebelly spinner using methods such as those outlined by Durand *et al*. [56].

Misassignment of samples to the wrong ETP subspecies could have occurred in the field, and it would impact our results. However, the effects would be mostly restricted to comparisons among ETP groups that have been analysed more thoroughly in other treatments [17,18]. Since misassignment was only likely in the ETP, where sample sizes are large, we do not expect possible sample misassignment in the ETP to impact the conclusions of this global study. If misassigned individuals were included in the study, our measure of population divergence among ETP groups based on allele frequency tests (*F*_ST_) would decrease. Misassignment would create sample outliers in the PCA results, which could also expand population ellipses. In the phylogeography analysis, misassigned samples could bring ETP lineages closer together and decrease branch support within the ETP, but would not impact the broader global phylogeny.

#### 4.1.4. Atlantic Ocean

Based on Davies [10], we would expect the Atlantic branch to be nested well within the clade including western Indian Ocean samples from Tanzania (labelled ZAN in figure 5). We were surprised by the placement of the Atlantic Ocean samples, close to the whitebelly spinner samples and near the core of the DAPC diversity of all samples. In addition, the deepest divergence within this clade was between the Atlantic Ocean and all other lineages. The Atlantic branch is again not where we would expect it to be given the vicariant step-stone hypotheses of Davies [10] and Perrin [11]. Instead, it is positioned at the base of this clade, well outside the clade including Tanzania. Andrews *et al*. [16] did include Atlantic specimens, and there was no evidence of connection between ETP spinners and Atlantic spinners in their results. Although we are unable to infer biogeographic events with this unrooted analysis, this position suggests a deeper relationship to the pantropical + dwarf clade and not a history of vicariant step-stoning west from the Pacific, to the Indian, and ultimately to the Atlantic Ocean. The ETP and Atlantic Ocean branches are the longest in this tree, so this position could be due to long-branch attraction. However, we doubt the relationship of these two lineages is a result of long-branch attraction, because if so, we might expect the Atlantic and ETP branches to be sister taxa within a clade, which they are not. Possible reasons for this Atlantic topology include: (i) that the age for spinner dolphin lineage has been underestimated and that the east Pacific basin was a much more formidable barrier to gene flow prior to the uplift of the Panamanian Isthmus, and/or (ii) the existence of historically super-abundant ETP + Atlantic populations whose high N_e_ slowed the effect of genetic drift over time.

Panamanian uplift occurred approximately 3.2 Ma [57], although newer evidence suggests it may be considerably older at 15–20 Ma [58]. The age of spinner dolphins, estimated using molecular clock techniques on a supermatrix consisting of 42 335 characters from mtDNA and nuDNA loci, is between 1.6 and 3.97 Ma (mean 2.75 Ma; [22]). From this estimate, we can infer that Panama would have been a significant barrier to spinner dolphins in the infancy of the species and should have led to significant drift between eastern tropical Pacific dolphins and northeastern Atlantic spinner dolphins. The only plausible reasons why this would not happen are ongoing or periodic pantropical gene flow (completely circumtropical gene flow) or incomplete lineage sorting. The former seems implausible given the barriers discussed. The latter scenario involves harbouring high genomic variation from a large ancestral population that has not yet drifted apart despite separation. This hypothesis has been invoked for a similar pattern found in dolphinfish (*Coryphaena hippurus*) [9]. These authors state that the lack of divergence by populations separated by the Isthmus of Panama reveals that genetic drift is weak in tropical pelagic species with high abundance.

Neither of these hypotheses adequately explains the positioning of the Atlantic genotypes near the whitebelly spinner group in genomic PC space. Of course, these explanations are not mutually exclusive.

Alternatively, if large populations harbouring diverse genomes continued to disperse around southern Africa and the east Pacific basin, it is possible that similarities could remain even with low levels of ongoing or sporadic gene flow. Atlantic spinner dolphins tend to be more pelagic (found exclusively in water of greater than 2000 m) and form larger groups than the island-associated central Pacific spinner dolphins [59]. They are also much lower in abundance in the Atlantic Ocean (11 971 in the Gulf of Mexico) than the eastern tropical Pacific [60] which could have led to lower heterozygosity, but would have also increased the genetic drift. Unfortunately, abundance estimates for the western north Atlantic coast of the United States are not available because sightings are rare along the continental shelf [59]. Therefore, it is difficult to tell if the greater Atlantic population would have been subject to a similar level of drift or if it would have been buffered from drift by large abundance. These are factors to consider as additional work brings clarity to our knowledge of ocean basin-level relationships in spinner dolphins.

Our ability to test the hypothesis of east–west pantropical stepwise dispersal patterns hinges on the evolutionary history of the Indian Ocean populations. If early stepwise dispersal occurred, leading to an Atlantic population in the North Atlantic and Caribbean, followed by extinction within the Indian Ocean, and subsequent recolonization events, the tight connection between Atlantic and Indian that anchor our null hypotheses of ocean-basin phylogeography might have been erased. Employing a method to root within-species trees, combined with the inclusion of additional samples for the eastern Atlantic and additional Indian Ocean localities, might help resolve this question.

#### 4.1.5. Hawaiian spinner dolphins

Despite being morphologically similar to other pantropical spinner dolphins, Hawaiian spinners are genetically unique and geographically isolated. The Hawaiian spinner genotypes occupied a very unique DAPC space and formed their own lineage within the pantropical + dwarf clade, the base of a well-supported Indo-Pacific clade. Although most closely related to this Indo-western Pacific core group of populations in the first eigenvalue, the second eigenvalue spread the Hawaiian genotypes away from the other populations. Mounting evidence, including those presented here, indicate that Hawaiian spinner dolphins are on a unique evolutionary trajectory and may warrant a unique subspecies.

Other lines of evidence [16,18,61] have shown that the Hawaiian population is divergent from other eastern Pacific populations—probably due to small population size and genetic drift. Our results from the DAPC in figure 3 are in agreement with these independent results. Given our findings (and those of others), this population should be minimally considered a distinct population segment. Taylor *et al*. [62] define a subspecies as ‘a population, or collection of populations, that appears to be a separately evolving lineage with discontinuities resulting from geography, ecological specialization, or other forces that restrict gene flow to the point that the population or collection of populations is diagnosably distinct’. The placement of Hawaiian Islands spinner dolphins in our phylogeographic and DAPC results show that they ‘appear to be a separately evolving lineage’, and combined with the fact they are comprised of multiple populations [61], careful consideration should be given to whether this population actually warrants subspecies status.

#### 4.1.6 Dwarf spinner dolphins

Opposite the ETP arm in our DAPC analysis (spread by the first eigenvalue) are the two populations of dwarf spinner dolphins: Indonesia and Northern Australia. The Indonesian dwarf spinner ellipse overlaps somewhat in genotype space with the Indo-western Pacific core populations, but has one individual midway between the core and the distant Northern Australia cluster. This could indicate a potential area of admixture between the western Pacific and Indian Ocean populations and the dwarf populations, or it could indicate a cline of diversity from these Indo-Pacific pantropical groups through the Indonesian Archipelago and south towards the genetically distant Australian dwarf spinners. The Australia dwarf spinners had the lowest observed heterozygosity compared to the other populations, indicating that this might be a small population. But the variance in this measure was high. We tested whether there was significant difference between the heterozygosity of the Indonesian population and the Australian population using a *T*-test. The two estimates were not significantly different at 95% confidence, indicating that our data do not show a significant difference in heterozygosity.

The largest point estimates of pairwise *F*_ST_ between populations of spinner dolphins involved the Australian population of dwarf spinner dolphins (all were greater than 0.1698). These results were corroborated by our DAPC analyses that indicated Australian dwarf spinners occupy a very different genomic PC space than the other subspecies and populations. In the pairwise comparison with the other dwarf population (Indonesia), *F*_ST_ was an order of magnitude larger (0.1698) than between the two morphologically distinct endemic ETP subspecies (CA and eastern: 0.0119). These results were unexpected given the morphological similarity between Australian and Indonesian dwarf spinner dolphins [14]. However, this pattern of strong genetic structure across the ‘Marine Wallace's Line’ (*sensu* [24]) has been shown in two recent studies of coastal cetaceans in this same area [63,64]. Collectively, these studies indicate that the Marine Wallace's Line is a significant biogeographic barrier despite the high dispersal abilities of marine mammals.

In our Bayesian species tree, the Indo-Pacific clade is split between the Indo-western Pacific group and a clade that links the dwarf spinner dolphin subspecies. Despite having relatively low support, this clade corresponds to the ‘Indo-western Pacific core’ from our DAPC analysis. Interestingly, within the dwarf spinner dolphin clade, the large differences in the DAPC analysis are not detected and the two dwarf spinner dolphin populations group together phylogenetically as suggested by morphological analyses [14].

In the broader sense, Barber *et al*. [24] proposed extending the terrestrial biogeographical break between western Indonesia and eastern Indonesia/Australia to the marine environment based on patterns of multiple reef-dwelling species. Recent work on the systematic relationships of small shallow-water dolphins highlighted the importance of this Marine Wallace's line as a biogeographic break. A new species of Irrawaddy dolphin (genus *Orcaella*) was described east of Wallace's line [63] and recently a new species of the genus *Sousa* was found to be separate from the Indo-Pacific *Sousa* species by a wide distributional gap that coincides with Wallace's Line [64].

Certainly, efforts should be made to estimate the abundance of these populations and better characterize them. Our sample set for northern Australia dwarf spinners was skewed heavily towards male dolphins (11 of 13 were males), but Y chromosome linked SNPs would represent only approximately 1/44th the SNPs—a very small fraction of the overall data. Therefore, this is unlikely to contribute to the uniqueness of this population. Given our very high significance in pairwise comparisons using *F*_ST_ and the distance in genotype space detected with DAPC, this Australian population of dwarf spinner dolphins (from the Timor Sea, north of Australia) could warrant a unique population of dwarf spinner or a unique subspecies. Perrin *et al*. [14] erected the subspecies *S. l. roseiventris* based on detailed morphometric analyses. They examined specimens from the Timor Sea and found them to be consistent with the dwarf spinner dolphins from the Gulf of Thailand. The only notable difference was northern Australia dwarf spinner dolphins had fewer vertebrae than those from the Gulf of Thailand. These authors did not examine dwarf spinner dolphins from Indonesia, but speculated that these animals inhabit shallow coastal waters of much of Southeast Asia. Further research effort should be applied to these areas to determine the geographical range and genetic diversity of dwarf spinner dolphins in Southeast Asia.

### 4.5. Spotted dolphins

Spotted dolphins were highly structured across their range (table 4); however, the major phylogeographic barriers differed from that of the spinner dolphins, with closer connections between western and eastern Pacific populations of the pantropical subspecies. *F*_ST_ point estimates showed a similar pattern to the spinner dolphins in that pairwise comparison of the coastal ETP subspecies with the offshore ETP population of pantropical spotted dolphins had reduced *F*_ST_ compared to pairwise comparisons between other global populations (although significantly different from zero). These results indicate a close relationship found in previous studies between the inshore/offshore subspecies in the ETP [17,21]. Moreover, almost all populations in this analysis had little to no overlap in the 67% ellipses, suggesting that these all represent unique isolated populations. The only exception was overlap from the Maldivian sample (*n* = 1) within the ellipse of the Guam/NMI population. It is difficult to draw conclusions based on one sample; therefore further studies should focus on expanding the sample of spotted dolphins in the Indian Ocean to test the uniqueness of the Maldivian population. The cluster of ellipses comprised from genotypes of spotted dolphins from Guam/MNI, Maldives and Indonesia is reminiscent of the ‘Indo-western Pacific core’ for spinner dolphins, with the exception of Indonesia, which in spinner dolphins was a more distant population. Hawaiian spotted dolphins spread out on the second eigenvalue, similar to spinner dolphins, to form a very distinct cluster. Again, this result in concert with the *F*_ST_ estimates, suggests that Hawaiian spotted dolphins may be on a separate evolutionary trajectory. Although also significantly different from zero, one of the lowest estimates was between Hawai‘i and Guam/NMI, suggesting similarities in allele frequency between these two Pacific island archipelagoes. *F*_ST_ comparisons involving the Indonesian and Hawaiian spotted dolphins had small sample sizes (*n* = 3 and 4, respectively) and future studies should include more. The largest *p*-value observed was in the comparison of these two populations (0.0359); it is possible this is large because of low sample sizes and not because of a lack of population structure between these two distant localities.

For many marine taxa, including the spinner dolphin, the east Pacific basin is a major biogeographic barrier [54]. Due to spatial proximity of the ETP spotted dolphin populations, the east Pacific basin should be a stronger biogeographic and habitat barrier [55] than the inshore–offshore divide. Instead, our results suggest that it is not as significant a barrier for spotted dolphins as the inshore–offshore divide in the eastern tropical Pacific. Moreover, our results support the current taxonomy as designated by morphology and genetics [17,21]. The results of our phylogeographic analysis for spotted dolphins indicate very close relationships among the Indo-Pacific populations. This could reflect recent divergences and possibly ongoing gene flow in this region. Subsequent studies should include the Atlantic pantropical spotted dolphin samples, and samples from northern Australia should be added to test the circumtropical biogeographic hypotheses in this species.

## 5. Conclusion

Both spinner and spotted dolphins appear to have tightly clustered Indo-western Pacific core relationships characterized by recent divergences, incomplete lineage sorting and/or ongoing gene flow.

In spinner dolphins, the east Pacific basin appears to be an important biogeographic barrier. Our results indicate deep separation between eastern Pacific clades and other conspecifics. The sister clade to the ETP group includes all other pantropical and dwarf populations. It is largely consistent with the morphology and current taxonomy with one issue; the dwarf spinner dolphin (*S. l. roseiventris*) is nested within the nominate pantropical subspecies (*S. l. longirostris*) making the latter non-monophyletic. Furthermore, our results do not support the connection between the Atlantic Ocean and the Indian Ocean via the South African species gate [11]. Instead, our results suggest the northwestern Atlantic Ocean population is a unique lineage, related to the pantropical spinner dolphin clade to the exclusion of the ETP clade, instead of nested within the pantropical spinner clade, where it would be expected via an Indian Ocean–Atlantic stepping stone model of dispersal. The pattern we observe could represent a very deep connection of a historically super-abundant population (prior to the uplift of Panama). The vicariance caused by the uplift should have caused the populations to drift apart, but high abundance and heterozygosity could have retained similarities. Alternatively, multiple extinctions and reinvasions could cause the pattern we see. We found the dwarf spinner dolphins from northern Australia to have a unique genomic signature. It is clear there is a connection phylogenetically with the dwarf spinner dolphins from Indonesia. However, based on our results, the northern Australia dwarf spinner dolphin should be considered a different population if not a different subspecies. This could be another example of the Marine Wallace's Line [24] in a shallow-water small cetacean [63,64].

Our analyses support the current taxonomic designation of the offshore spotted dolphin in the eastern tropical pacific as part of the pantropical subspecies. Although we were not able to test relationships with Atlantic populations of spotted dolphins, our results support the current taxonomy and indicate that, in contrast to the phylogeographic pattern in spinner dolphins, the inshore–offshore boundary in the ETP is stronger than the ecological and spatial divide of the pelagic expanse of the eastern Pacific.

## Supplementary Material

Leslie and Morin_Supplementary Figures

## Footnotes

1 Remco Bouckaert – Research Fellow, Dept. of Computer Science, The University of Auckland, Auckland, NZ. April 2014 http://tinyurl.com/j7uwq83

## Permission to carry out fieldwork

Scientific Research Permits were obtained by NOAA-NMFS under US Marine Mammal Protection act permits to SWFSC, PIFSC, and Cascadia Research Collective. Many of these samples were collected in international waters. Written permission was secured prior to conducting scientific research in non-US or non-international waters.

## Data accessibility

Filtered datasets and R-code used for analyses are available as .rdata environment and .r script file on Dryad Digital Repository: (http://dx.doi.org/10.5061/dryad.h9q13) [35].
