# Supplementary material for: Structure and phylogeography of two tropical predators, spinner (Stenella longirostris) and pantropical spotted (S. attenuata) dolphins, from SNP data
Source: R Soc Open Sci. 2018 Apr 25;5(4):171615. doi: 10.1098/rsos.171615 (PMC5936904; doi:10.1098/rsos.171615)
Supplement: Leslie and Morin_Supplementary Figures [file rsos171615supp1.docx]

**Figure S1.** Cumulative genomic variation explained by principle components 1 through 120 in the global spinner dolphin dataset.

**Figure S2.** Cumulative genomic variation explained by principle components 1 through 70 in the spotted dolphin dataset.

**Figure S3. Alpha (or a-score) optimization for the spinner dolphin dataset – spline interpolation for PCs 1 through 39.** Box and whiskers show the overall mean and variance of individual population a-score for each of the PCs representing the global spinner dolphin dataset based on 10 simulations. Seven PCs had the highest estimated mean a-score for the dataset and was chosen as the “optimal” is subsequent analyses of DAPC. Thirty-nine PCs was also chosen as a “highest recommended” number of PCs (the number of PCs should be <N/3).

**Figure S4. Alpha (or a-score) optimization – spline interpolation for PCs 1 through 25 for spotted dolphin data.** Box and whiskers show the overall mean and diversity of individual population a-score for each of the PCs representing the ETP spotted dolphin dataset based on 10 simulations. Seven PCs had the highest estimated mean a-score for the dataset and was chosen as the “optimal” is subsequent analyses of DAPC. Twenty-five PCs was also chosen as a “highest recommended” number of PCs (the number of PCs should be <N/3).

**Table S1.** List of spinner dolphin samples subset from the total dataset and used for global phylogeographic analyses in *SNAPP*.

| LabID | subspecies | Slon_GBL_22 | Latitude | Longitude | SEX |
| --- | --- | --- | --- | --- | --- |
| 367 | roseiventris | DWF_AUS | -12.633333 | 126.4 | M |
| 372 | roseiventris | DWF_AUS | -12.6 | 127.3 | M |
| 392 | longirostris | PHIL | 8.85 | 123.116666 | F |
| 400 | longirostris | PHIL | 8.85 | 123.116666 | U |
| 462 | longirostris | ATL | 36.383333 | -75.816666 | M |
| 2138 | longirostris | WB | 8.6 | -138.216666 | M |
| 2757 | longirostris | ATL | 28.666666 | -87.766666 | M |
| 4095 | longirostris | WB | 8.6 | -138.216666 | M |
| 7185 | longirostris | HI | 20 | -155.833333 | M |
| 7202 | longirostris | HI | 19.666666 | -156.083333 | M |
| 9847 | longirostris | MALDIVES | 4.766666 | 73.5 | F |
| 9854 | longirostris | MALDIVES | 6.933333 | 73.233333 | M |
| 11664 | centroamericana | CA | 15.866666 | -94.833333 | F |
| 24928 | orientalis | EAST | 10.2 | -98.533333 | F |
| 38017 | orientalis | TM | 23.633333 | -107.383333 | F |
| 38018 | orientalis | TM | 22.25 | -106.4 | F |
| 38050 | orientalis | EAST | 21.45 | -106.066666 | M |
| 38191 | centroamericana | CA | 12.583333 | -88.533333 | M |
| 47281 | longirostris | ZAN | -4.866666 | 39.65 | U |
| 47283 | longirostris | ZAN | -4.866666 | 39.65 | U |
| 79908 | roesiventris | DWF_INDO | 2.266666 | 118.3 | U |
| 79923 | roseiventris | DWF_INDO | 1.3 | 118.75 | U |

**Table S2.** List of spotted dolphin (*Stenella attenuata*) samples subset from the total dataset and used for global phylogeographic analyses in SNAPP.

| LabID | subspecies | Satt_GBL_reduced | Latitude | Longitude | SEX |
| --- | --- | --- | --- | --- | --- |
| 130 | attenuata | ETP.Off | 14 | -96.083333 | U |
| 2081 | attenuata | ETP.Off | 13.283333 | -128.766666 | F |
| 2092 | attenuata | ETP.Off | 12.8 | -131.183333 | F |
| 9860 | attenuata | MAL | 7.183333 | 73 | U |
| 11381 | graffmani | Coastal | 16.45 | -99.3 | M |
| 11921 | graffmani | Coastal | 11.466666 | -86.566666 | F |
| 11931 | graffmani | Coastal | 9.433333 | -85.466666 | M |
| 11950 | graffmani | Coastal | 7.65 | -82.333333 | M |
| 12039 | graffmani | Coastal | 0.183333 | -80.5 | F |
| 24047 | attenuata | ETP.Off | 5.866666 | -90.066666 | M |
| 30485 | attenuata | HI | 21.7 | -157.616666 | F |
| 55197 | attenuata | HI | 19.283333 | -156 | F |
| 56750 | attenuata | HI | NA | NA | M |
| 75657 | attenuata | HI | 19.4 | -156.033333 | M |
| 79906 | attenuata | INDO | 2.3 | 118.466666 | U |
| 79912 | attenuata | INDO | 2.183333 | 118.533333 | U |
| 79915 | attenuata | INDO | 2.366666 | 118.433333 | U |
| 108200 | attenuata | Guam | 13.7 | 144.816666 | U |
| 108201 | attenuata | Guam | 13.7 | 144.816666 | U |
| 116842 | attenuata | NMI | 14.126452 | 145.059145 | U |
| 116844 | attenuata | NMI | 14.127214 | 145.076016 | U |
| 116849 | attenuata | NMI | 14.067994 | 145.209951 | U |

**
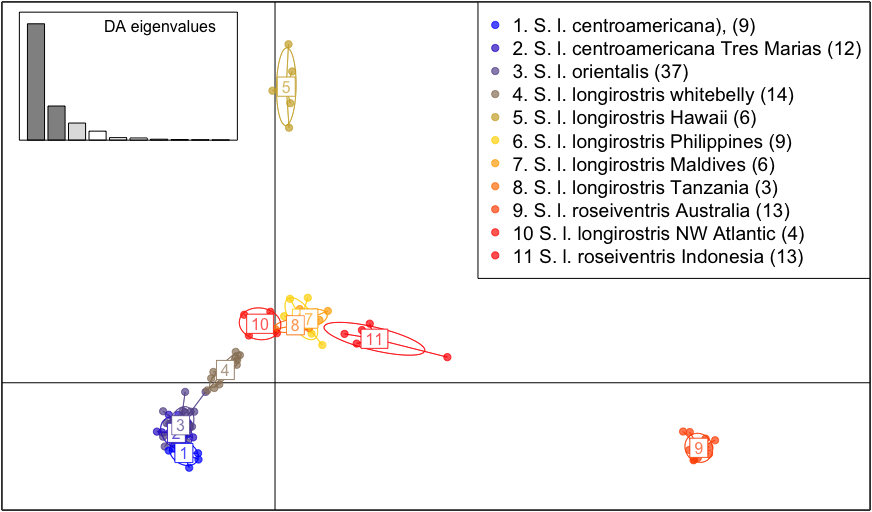
**

**Figure S5. Genomic variation across individuals and populations of spinner** **dolphins using 39 principle components and 3 discriminant analyses:** Scatter plot of individuals based on the first two eigenvalues of the Discriminant Analysis of Principle Components (DAPC). Inset shows the amount of variation represented by the DA eigenvalues. Ellipses represent 67% of the variation for each population. Group 1 is Central American spinner (*S. l. centroamericana*), Group 2 is from the Tres Marias Islands, Group 3 is the eastern spinner (*S. l. orientalis*), Group 4 is the whitebelly spinner (*S. l. longirostris*), Group 5 is the Hawaii Islands (*S. l. longirostris*), Group 6 is samples from the Philippines (*S. l. longirostris*), Group 7 are samples from the Maldivies (*S. l. longirostris*), Group 8 is Indian Ocean pantropical spinner dolphin (*S. l. longirostris*), Group 9 is samples of the dwarf subspecies (*S. l. roseiventris*) from Australia, Group 10 is Atlantic pantropical spinner dolphin (*S. l. longirostris*), Group 11 is samples of the dwarf subspecies (*S. l. roseiventris*) from Indonesia.

**
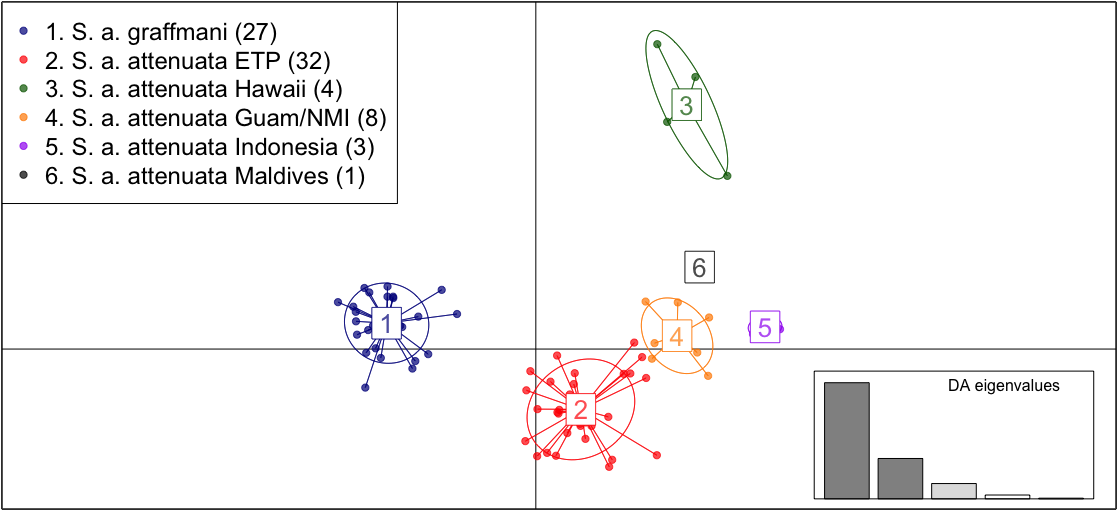
**

**Figure S6. Genomic variation across individuals and populations of spotted** **dolphins using 25 principle components and three discriminant analyses:** Scatter plot of individuals based on the first two eigenvalues of the Discriminant Analysis of Principle Components (DAPC). Ellipses represent 67% of the variation for each population. Inset shows the amount of variation represented by the DA eigenvalues. Population 1. is the Coastal ETP subspecies (*S. a. graffmani*); all others are *S. a. attenuata* populations. Group 2 is Offshore ETP, Group 3 is Hawaii, Group 4 is Guam/NMI, Group 5 is Indonesia, Group 6 is Maldives. Maldives is represented by only one sample.

**a)**

**b)**

**Figure S7. Trace (a) and density distribution (b) for spinner dolphin Bayesian phylogeographic analysis.** MCMC run for 401,000 chains.

**a)**

**b)**

**Figure S8. Trace (a) and density distribution (b) for spotted dolphin Bayesian phylogeographic analysis.** MCMC run for 1M chains.
